# Emotion regulation, optimism and quality of life among Gastric Ulcer Patients

**DOI:** 10.12669/pjms.37.4.3894

**Published:** 2021

**Authors:** Amina Muazzam, Nida Ali, Yasmeen Niazi, Naima Hassan

**Affiliations:** 1 Dr. Amina Muazzam Tenured Professor, Department of Applied Psychology, Lahore College for Women University (LCWU), Lahore, Pakistan; 2 Nida Ali Department of Applied Psychology, Lahore College for Women University (LCWU), Lahore, Pakistan; 3 Yasmeen Niazi Department of Applied Psychology, Lahore College for Women University (LCWU), Lahore, Pakistan; 4 Dr. Naima Hassan Assistant Professor, Department of Psychology, Virtual University of Pakistan, Lahore, Pakistan

**Keywords:** Emotion regulation, Optimism, Quality of life, Gastric ulcer

## Abstract

**Objective:**

The study was aimed to investigate the association among emotion regulation, optimism and quality of life among gastric ulcer patients.

**Methods:**

The Cross-sectional study was conducted in public sector hospitals of Lahore, during January-June 2017. Sample of study was comprised of 100 patients diagnosed with gastric ulcer, aged 25-55 years, selected through non-probability purposive sampling technique. Demographic information sheet, Emotion Regulation Scale by John, Gross 2003, Life Orientation Test-Revised by Sheer, Carver 2002, Quality of Life Enjoyment and Satisfaction Questionnaire by Endicott 1993 were used for data collection. SPSS 21 version was used for data analysis.

**Results:**

Out of total 100 participants 41 (41%) were men and 59 (59%) were women, with mean age of (M= 44.89, SD= 7.99). There was significant positive correlation among emotion regulation, optimism and quality of life (p<0.01). Optimism and emotion regulation were observed as significant predictors of quality of life (p<0.01). Significant gender differences were found in emotion regulation (p< 0.01), optimism (p< 0.01) and quality of life (p<0.01), with men scoring higher as compared to women. One way ANOVA showed significant differences between emotion regulation, optimism and quality of life among different age groups of gastric ulcer patients (p< 0.01).

**Conclusion:**

Quality of life of gastric ulcer patients can be greatly improved by effectively using emotion regulation strategies and optimistic approach.

## INTRODUCTION

Digestive system diseases are remarkably common throughout the world. Peptic ulcers are reported as the leading cause of hospitalization due to upper gastrointestinal bleeding. Peptic ulcer is a sub-mucosal injury in the digestive tract that most commonly occurs in the stomach and duodenum. Infection with *H. Pylori*, excessive use of NSAIDs and stress are the main cause of gastrointestinal ulcers. The incidence of gastric ulcers augments with age and is more prevalent in females (60%) than males (40%). Globally most prevalent type of gastrointestinal ulcers is gastric ulcer, affecting around 5-10% of people annually.^1^

Emotion regulation is a set of cognitive processes that determine the type of emotional response and also how a person will experience and express these emotions. The most widely studied and commonly employed emotion regulation strategies are expressive suppression and cognitive reappraisal. Expressive suppression is inhibition of the expression of an emotional response, while cognitive reappraisal is the reinterpretation of an emotional situation to modify its impact.^2^ Peptic ulcer patients experience more emotional and psychiatric problems that lead to dysfunctional emotional regulation as compared to healthy individuals.^3^ It is reported that the use of adaptive strategies like cognitive reappraisal, and reducing maladaptive strategies like expressive suppression could be effective for patients with gastrointestinal disorders.^4^ People dealing with emotional deregulation, maladjustment, unfavorable life events, poor sleep quality,^5^ depression and anxiety are considerably more at risk of developing the peptic ulcers.^6^

Optimism is the generalized expectancy of the positive outcome.^7^ Optimism is positively related to better mental and physical health by promoting healthy lifestyles and adaptive behaviors associated with problem-solving ability and more effective elaboration of negative information.^7^ Optimistic people tend to pay attention towards diverse aspects of a situation that in turn help them bounce back more efficiently from stressful situations. Better psychological resources like hope, optimism and self-esteem have a positive influence on health and quality of life.^8^

Quality of life is the insight of a person about their life, culture and value system in which they are living and in association with the goals, expectations and concerns.^9^ Although peptic ulcers are not life-threatening but still, they deteriorate the quality of life of patients by restricting their ability to actively participate in their daily life activities, and in turn pose a significant burden to the health care system.^10^ Use of proper treatment interventions and preventive strategies were found to have a positive impact on the quality of life in peptic ulcer patients.^11^

Our study aimed to assess the association of gastric ulcer with emotion regulation, optimism and quality of life. The predictive relationship between emotion regulation, optimism and quality of life was also investigated. Additionally, we tried to identify the difference in the level of emotion regulation, optimism and quality of life among both genders and along with the different age groups.

## METHODS

The cross-sectional study was conducted between January 2017 to June 2017, in Lahore. The sample of the study consisted of 100 adults; 25-55 years old with gastric ulcers. Among participants 59 (59%) were women and 41 (41%) were men, 30 (30%) were educated and 40 (40%) were uneducated, 29 (29%) belonged to upper middle class, 41 (41%) lower middle class and 30 (30%) belonged to lower class, and 50 (50%) were employed and 50 (50%) were unemployed. Sample was selected through non-probability purposive sampling. Only people diagnosed with gastric ulcers by the physician and in the mentioned age range were selected. Before data collection, informed consent was signed by the participants and they were assured about the confidentiality of their information. Basic details regarding gender, age, marital status, family history of the disease and other necessary details were obtained through a demographic sheet. All the patients included in the study were clinically diagnosed with gastric ulcers however the patients with any other comorbid condition were excluded from the sample.

Emotion regulation scale was used to measure the emotional state of individuals. This is a self-reported 10 item scale with two subscales;(i) cognitive reappraisal consisted of item no 1,3,5,7,8,10 and (ii) expressive suppression consisted of item no 2,4,6,9. Responses were rated on 7 points likert scale (1=strongly disagree to 7=strongly agree). The reliability of both subscales was reported as .89-.90 for cognitive reappraisal items and .76-.80 for expressive suppression items.^12^

LOS was used to measure dispositional optimism is a short form 10 item scale, with two subscales (i) Optimism and (ii) Pessimism. The optimism subscale consists of items 1, 2, 4,5,6,8, 10 and the pessimism subscale consists of items 3, 7, 9. The questionnaire comprised of 6 scale items and 4 filler items, while the internal reliability of the scale was reported as .78. Likert point format (0=strongly disagree to 4=strongly agree) was used for grading of responses.^13^

Quality of Life Enjoyment and Satisfaction Questionnaire was used to evaluate the quality of life of patients. It is a 15-item scale graded on 5 points likert format (1=very poor to 5=very good). Moreover, internal consistency of the scale was reported as 0.93.^14^

SPSS 21 version was used for data analysis. Descriptive statistics was carried out for the analysis of basic variables. Whereas Independent sample t-test, linear regression analysis and one-way analysis of variance were conducted to test the study hypotheses. Ethical clearance was obtained from the ethical and research committee of the Lahore College for Women University, Lahore, with IRB number 2738 and issue date of 02-11-2020.

## RESULTS

Among the 100 participants, 41 (41%) were men and 59 (59%) were women. The overall mean age was 44.89±7.99. The majority 65 (65%) were having a family history of gastric ulcers. Linear regression analysis showed that emotion regulation (p<0.01) and optimism (p<0.01) were significant predictors of quality of life (Table-I). Independent sample t-test revealed significant gender differences, where men scored higher in emotion regulation (p<0.01), optimism (p<0.01) and quality of life (p<0.05) as compared to women (Table-II).

**Table-I:** Linear Regression Analysis predicting Quality of life from reappraisal, suppression and optimism (n=100).

*Predictor Variable*	*B*	*SE*	*Β*	*T*	*R^2^*	*P*	*Dependent Variable*
Optimism	0.67	0.11	0.50**	5.76	0.25	0.00	Quality of Life
RA(ER)	0.11	0.08	0.18**	1.32	0.02	0.00	
SP(ER)	0.10	0.10	0.14**	1.00	0.03	0.00	

***Note:*** p<0.01.

**Table-II:** Summary of Independent Sample t-test (n=100).

*Variable*	*Men*		*Women*					

	*(n= 41)*		*(n = 59)*					
	*M*	*SD*	*M*	*SD*	*Df*	*T*	*P*	*Cohen’s d*
RA(ER)	23.5	7.63	25.5	8.30	98	1.74	0.001*	0.40
SP(ER)	17.7	6.62	15.4	6.54	98	-1.20	0.001*	0.35
OPT	14.3	3.43	12.01	3.26	98	3.46**	0.001*	0.70
QOL	40.9	5.0	38.8	4.3	98	2.17	0.032**	0.45

***Note:*** RA=reappraisal, SP=suppression OPT= optimism, QOL=quality of life,

* p<0.01,

** p<0.05 Independent sample t-test revealed significant gender differences in Reappraisal (p<0.01), Suppression (<0.01), optimism (p<0.01) and quality of life (p<0.05), where males scored higher for each variable as compared to females (Table-II).

One-way analysis of variance showed significant differences between emotion regulation (reappraisal and suppression), optimism and quality of life among different age groups of gastric ulcer patients (p<0.01). Young and middle age patients experienced better quality of life and a relatively high level of optimism as compared to the older age group of gastric ulcer patients. Results also suggested that unlike the older age group, young and middle age patients tend to regulate their emotions through reappraisal than suppression (Table-III).

**Table-III:** Summary of One-way Analysis of Variance (ANOVA).

	*25-34(n=33)*		*35-44(n=33)*		*45-55(n=34)*	

	*M*	*SD*	*M*	*SD*	*M*	*SD*	*F*	*P*
QOL	53.06	11.1	39.15	10.4	36.26	9.65	24.68	0.000**
OPT	16.72	3.54	13.33	4.58	9.44	3.80	27.84	0.000**
RA(ER)	32.72	4.76	24.54	5.05	17.11	4.92	84.37	0.000**
SP(ER)	9.15	3.71	16.84	3.27	22.94	3.45	131.28	0.000**

***Note:*** ER= emotion regulation, OPT= optimism, QOL= quality of life, Between group *df*=2, within group *df*=97, total *df*=99,

** p<0.01.

## DISCUSSION

In this study, we found a close association between emotion regulation, optimism and quality of life among gastric ulcer patients. Very few studies have been conducted on the association of these variables with gastric ulcers. Our study revealed that optimism and emotion regulation are predictors of quality of life among gastric ulcer patients. People who tend to pay more attention to emotional response of their disease and contemplate more on the sadness, report low quality of life as compared to healthy individuals.^15^ Psychological factors can contribute to 30% to 35% of ulcers, even in the absence of H. pylori infection. It has been debated that personality disturbances may be significantly linked to the elevated stomach ulcer risks.^8^ There is a strong association between pessimism, psychosocial factors, quality of life, and peptic ulcer disease.^16^ While adopting a positive emotional attitude enable people with peptic ulcer improve their quality of life, and significantly reduce distress, anxiety and depression.^17^ Emotion regulation capacities (like positive reappraisal) can play a unique role in predicting particular aspects of adjustment among individuals with chronic illness. Optimism motivates people to take proactive measures to protect their health and prosper in the middle of the adverse situation.^18^ Optimistic people show more compliance towards treatment regimens and are further benefited in terms of quality of life.^19^

Significant gender differences were observed where men scored higher in emotion regulation, optimism and quality of life as compared to women. The difference in the quality of life among males and females with peptic ulcer is a common finding, men tend to have significantly better quality of life and mental health as compared to women.^8^ Generally women more intensely experience the emotions related to their disease, and they also feel it difficult to control their negative thoughts regarding the illness,^7^ which in turn lead to the reduced quality of life.^11^ Gender differences in emotion regulation are mainly because young women experience higher levels of rumination and worry than men. These negative thoughts may lead to greater feelings of hopelessness as well decreased quality of life.^20^

The present study also revealed that young and middle-aged group of patients showed relatively better quality of life and a greater level of optimism than older age gastric ulcer patients. Although gastric ulcer is found in all age groups, its onset is more common after 30 years and is influencing older people more often as compared to younger people.^17^ It is reported previously that quality of life deteriorates over time in gastric ulcer patients,^21^ various factors like duration and intensity of disease are associated with the reduced quality of life in older patients.^22^ As Increasing age is related to the decrease in physical activity and an increase in the mental activity, older women with gastrointestinal disorders are generally more disadvantaged in daily life activities than men,^15^ resulting in the decline in their quality of life. Peptic ulcer disease is associated with the high cost of health care services and it greatly impairs the well-being and health related quality of life of the older patients.^23^ Significant differences were found regarding the age and the selection of emotion regulation strategies. Existing literature supporting these findings reported that suppression is a frequently practiced type of emotion regulation in the older age group.^24^ Studies indicate that young and middle aged group of patients tend to regulate their emotions through reappraisal. In comparison to the suppression the reappraisal strategy significantly predict both the subjective and objective quality of life.^17^ It is reported previously that expressive suppression is associated to the elevation of the symptoms of distress and anxiety and reduced quality of life in stomach ulcer patients.^25^

### Limitations of the study

Participants were selected from a few hospitals of Lahore only, for this reason the results may not be the true representative of the general population. To resolve the issue community studies with a large sample size should be conducted. The relationship of gastric ulcer and its psychological correlates involving the changes in environmental and personal factors are needed to be studied.

## CONCLUSION

This study revealed that gastric ulcer patients who are optimistic tend to adopt better coping strategies and ultimately have a better quality of life. Moreover, young male participants reported a relatively higher level of optimism and emotion regulation leading to improved quality of life as compared to older ones.

### Implications

The overall finding of current study emphasizes the beneficial role of emotion regulation and optimism against the quality of life in gastric ulcer patients. Women are more vulnerable to negative emotions, stress and reduced optimism as compared to men. This study will eventually enrich the existing efforts that aimed at preventing the negative psychological impact of gastric ulcer.

### Authors Contribution:

**AM & NA:** Conceptualized the idea and prepared the manuscript.

**YN:** Edited draft and helped in data collection.

**AM & NH:** Did the statistical analysis.

**AM, NA, YN & NH:** Did review and final approval of the manuscript.

**NA:** She is responsible and accountable for the accuracy or integrity of the work.

## References

[1] Bibi F, Alvi SA, Sawan SA, Yasir, M (2017). Detection and Genotyping of Helicobacter pylori among Gastric ulcer and Cancer Patients from Saudi Arabia. Pak J Med Sci.

[2] Gross JJ (1998). Antecedent- and response-focused emotion regulation:divergent consequences for experience, expression, and physiology. J Pers Soc Psychol.

[3] Faramarzi M, Kheirkhah F, Shokri-Shirvani J, Mosavi S, Zarini S (2014). Psychological factors in patients with peptic ulcer &functional dyspepsia. Caspian J Intern Med.

[4] Mazaheri M, Afshar H, Nikneshan S, Adibi P (2016). Cognitive emotion regulation strategies in patients with functional dyspepsia and healthy controls - A comparative study. Adv Biomed Res.

[5] Levenstein S, George K, Margot S (2007). Psychological predictors of peptic ulcer incidence in the Alamdea County Study. J Clin Gastroenterol.

[6] Havens JM, Castillo-Angeles M, Nitzschke SL, Salim A (2018). Disparities in peptic ulcer disease:A nationwide study. Am J Surg.

[7] Shamim A, Muazzam A (2018). Positive Emotions and Compliance as Predictors in the Management of Type II Diabetes. Pak J Soc Clin Psychol.

[8] Wen Z, Li X, Lu Q, Brunson J, Zhao M, Tan J (2014). Health related quality of life in patients with chronic gastritis and peptic ulcer and factors with impact:A longitudinal study. BMC Gastroenterol.

[9] (2014). WHOQOL:Measuring Quality of Life.

[10] Hassan N, Muazzam A (2019). Development and Validation of Scale for Neuro-Psychological and Physiological Side Effects of Interferon Therapy (NPPSI) in HCV Patient. J Soc Clin Psychol.

[11] Zuo Y, Wang S, Cui X, Lv H (2018). Therapeutic Endoscopy in Combination with Quadruple Therapy in Treating Bleeding Caused by Gastric Ulcer. Pak J Med Sci.

[12] Preece D, Becerra R, Robinson K, Gross J (2020). The Emotion Regulation Questionnaire:Psychometric Properties in General Community Samples. J Pers Assess.

[13] Scheier MF, Carver CS, Bridges MW (1994). Distinguishing optimism from neuroticism (and trait anxiety, self-mastery, and self-esteem):A re-evaluation of the Life Orientation Test. J Pers Soc Psychol.

[14] Stevanovic D (2011). Quality of Life Enjoyment and Satisfaction Questionnaire-short form for quality of life assessments in clinical practice:A psychometric study. J Psychiatr Ment Health Nurs.

[15] Chung KT, Shelat VG (2017). Perforated peptic ulcer - an update. World J Gastro Surg.

[16] Feldman M, Walker P, Green JL, Weingarden K (1986). Life events stress and psychosocial factors in men with peptic ulcer disease. A multidimensional case-controlled study. Gastroenterol.

[17] Baghianimoghadam MH, Mohamadi S, Baghianimoghadam M, Falahi A, Roghani HS (2011). Survey on Quality of Life related factors in patients with Peptic Ulcer based on PRECEDE Model in Yazd, Iran. J Med Life.

[18] Conversano C, Rotondo A, Lensi E, Della-Vista O, Arpone F, Reda MA (2010). Optimism and its impact on mental and physical well-being. Clin Prac Epidemiology Ment Health.

[19] Fisher S (1994). Psychological aspects of the treatment of duodenal ulcer. Stress Health.

[20] Brummer L, Stopa L, Bucks R (2013). The Influence of Age on Emotion Regulation Strategies and Psychological Distress. Behav Cogn Psychother.

[21] Niazi Y, Ejaz B, Muazzam A (2020). Impact of hearing impairment on psychological distress and subjective well-being in older adults. Pak J Med Sci.

[22] Roy S (2016). Clinical Study of Peptic Ulcer Disease. Asian J Biomed Pharm.

[23] Hafez AA, Tavassoli E, Hasanzadeh A, Reisi M, Javadzade SH, Imanzad M (2013). Quality of life in peptic ulcer patients referring to Al-Zahra hospital of Isfahan, Iran. Gastroenterol Hepatol Bed Bench.

[24] Jorgensen RS, Johnson TB, Kolodziej ME, Schreer G (1996). Elevated Blood Pressure and Personality:A Meta-Analytic Review. Psychological Bulletin.

[25] Schiavon CC, Marchetti E, Gurgel LG, Busnello FM, Reppold CT (2017). Optimism and Hope in Chronic Disease:A systematic review. Front Psychol.

